# Exploration of Chinese cultural communication mode based on the Internet of Things and mobile multimedia technology

**DOI:** 10.7717/peerj-cs.1330

**Published:** 2023-04-18

**Authors:** Dan Xie, Chao Yin

**Affiliations:** 1 General Department, Xi’an Traffic Engineering Institute, Xi’an, Shaanxi, China; 2 University of Technology MARA, Selangor, Shah Anam, Malaysia; 3 School of History and Culture, Shaanxi Normal University, Xi’an, Shaanxi, China; 4 Xi’an Tie Yi Middle School, Xi’an, Shaanxi, China

**Keywords:** Shaanxi culture, Shadow play image, ResNet50, Iot, Image retrieval

## Abstract

Image retrieval technology has emerged as a popular research area of China’s development of cultural digital image dissemination and creative creation with the growth of the Internet and the digital information age. This study uses the shadow image in Shaanxi culture as the research object, suggests a shadow image retrieval model based on CBAM-ResNet50, and implements it in the IoT system to achieve more effective deep-level cultural information retrieval. First, ResNet50 is paired with an attention mechanism to enhance the network’s capacity to extract sophisticated semantic characteristics. The second step is configuring the IoT system’s picture acquisition, processing, and output modules. The image processing module incorporates the CBAM-ResNet50 network to provide intelligent and effective shadow play picture retrieval. The experiment results show that shadow plays on GPU can retrieve images at a millisecond level. Both the first image and the first six photographs may be accurately retrieved, with a retrieval accuracy of 92.5 percent for the first image. This effectively communicates Chinese culture and makes it possible to retrieve detailed shadow-play images.

## Introduction

An ancient form of art with a more than 2,000-year history, the Shaanxi shadow play, possesses distinctive aesthetic characteristics and traditional cultural meanings of China (Xu, 2022). Bionic approaches present shadow play patterns, which can be broadly divided into three groups. Terrestrial life, bird life, aquatic life, and wave motion. These patterns are exquisite in shape, rich in connotation and highly decorative, and have been widely used in packaging, clothing and household goods (Sabine, 2021). With the development of the times, the forms of entertainment have become more diversified, and shadow play art is facing the crisis of withdrawing from the historical stage. The research on shadow play patterns mainly focuses on artistic analysis, application, inheritance and protection, while the research on pattern retrieval technology is less involved (Lu, 2022). Therefore, the application of computer image retrieval technology to the study of shadow play patterns is helpful further to explore its internal culture and more objective redesign application and to expand a new direction for the spread of art and culture in the information age (Hussain et al., 2022).

Early image retrieval technologies are mainly divided into two categories (Antima & Rajendra, 2017): text-based image retrieval (TBIR) and content-based image retrieval (CBIR). TBIR uses text annotation to describe keywords of image information. When searching, the system searches for the matching image according to the query keyword provided by the user and returns it to the user (Guo & Sun, 2022). TBIR relies on subjective manual labeling, which will reduce retrieval accuracy. With the increase in image data, obtaining the corresponding label information is undoubtedly time-consuming and labor-intensive, and relying entirely on manual labeling will be inefficient. In addition, preserving annotation information also takes up a lot of storage space. Due to the above disadvantages, content-based image retrieval technology has gradually become a research hotspot and developed rapidly (Qu & Ren, 2022). Among them, CBIR technology uses manual extraction of visual features such as edge, texture and color of images and searches images through image feature matching and similarity measurement (Kaur, Lal & Kaur, 2020). Compared with TBIR technology, it saves the time of text annotation and overcomes the subjectivity of manual annotation, thus improving the efficiency and accuracy of retrieval. However, there is a semantic gap between the low-level visual features of images extracted by manual parts and the high-level semantic concepts, which hinders the progress and development of CBIR technology (Amin & Qureshi, 2020).

The deep learning algorithm represented by a convolutional neural network (CNN) marks a new step in computer vision research (Wang, Wang & Sun, 2022). CNN has robust learning and generalization abilities and can extract image texture and high-level semantic information using deep learning methods. Therefore, feature learning based on convolutional neural networks gradually replaces traditional hand-designed features and combining deep learning with content-based image retrieval can significantly improve image retrieval (Aman & Payal, 2020). For example, Ye et al. (2020) used the fine-tuned CNN model to extract image features and assign weights to retrieve image categories. Radenovic, Tolias & Chum (2019) proposed a trainable generalized-mean pooling (GeM) layer to improve the retrieval performance. Bhandi & Devi (2019) learned features from the pre-training model of the visual geometry group (VGG) and residual network, and created feature fusion to improve image retrieval accuracy by adjusting, perfecting, or fusing network features. Guo et al. (2019) proposed a local attention network to obtain the details of local features to achieve accurate image retrieval. Noh et al. (2017) suggested a DEep Local Features (DELF), which uses an attention mechanism to extract dense local features and obtain more accurate feature retrieval. Nie et al. (2019) proposed an attention based image retrieval (ABIR) method without boundary boxes, combined with CNN to realize image retrieval. However, with the increasing scale of image data, both traditional manual features and depth features can’t avoid the dimension disaster, which will bring tremendous pressure to storage and calculation and then affect the retrieval efficiency.

With the advancement of the intelligent process of information systems, the requirements for image retrieval technology are not only limited to improving the accuracy of image retrieval but also need to allocate and optimize the required resources rationally. Under the guidance of such requirements, the Internet of Things (IoT) system has become the research hot (Gaurav & Atulya, 2022). The IoT identifies and manages data resources by connecting modules with modules through identification and intelligent sensing technology. The following are the primary components of its key technologies at the moment. The initial perception technology. The Internet of Things is an information network system that relies on sensors. High-quality sensors can guarantee real-time, low-cost, and low-power data collecting. It can gather and calibrate the pertinent data through a single electrical interface, which can be applied to all system control linkages. The technology of energy supply, second. The energy issue is one of the IoT’s limits because of how many terminal nodes there are and how it connects the network and application levels. With energy storage and energy collection technology, the design of Internet of Things (IoT) devices and nodes can significantly lower energy usage and even better manage power consumption.

Third, gateway and communication technologies. The IoT contains a lot of terminal nodes, and that number is continually increasing. Communication and gateway technology can integrate wired and wireless communication, thus achieving the collaborative construction of local and wide areas, communication and sensing—fourth data fusion. In the face of the increasing data volume, data fusion has become a key means to solve the problem of data processing and storage in the IoT. It can reduce broadband occupation and improve response speed by establishing data autonomous settlement and application fusion methods. Embedding the image retrieval model into the IoT system can realize remote, reliable, safe, cooperative and intelligent model regulation through various modules in real time (Xu et al., 2022). Therefore, deploying the image retrieval model to the IoT system can better provide its law and application, reduce the training and testing time, and complete more intelligent and efficient image retrieval tasks.

This research suggests a CBAM-ResNet50 image retrieval model that retrieves shadow play images accurately and further advances cultural communication. It deploys it to the IOT system to accomplish accurate and efficient shadow play image segmentation. The contributions are as follows:

(1) using residual network ResNet50, combined with convolutional block attention module (CBAM), a CBAM-ResNet50 shadow image retrieval model is constructed.

(2) Based on the weighted sum of mean square error and Pearson distance, the loss function under double constraints is established, and the retrieval error is calculated to improve the retrieval ability of the network further.

(3) Deploy the CBAM-ResNet50 shadow image retrieval model to the IoT system to achieve more efficient shadow image retrieval.

The structure of this article is as follows: Section 2 introduces the shadow image retrieval model based on CBAM-ResNet50; Section 3 introduces the deployment of the Internet of Things system. Section 4 shows the retrieval performance of this model through experiments. Section 5 summarizes and looks forward to the content of this article.

## Shadow Image Retrieval Model Based on CBAM-ResNet50

**Figure 1:**
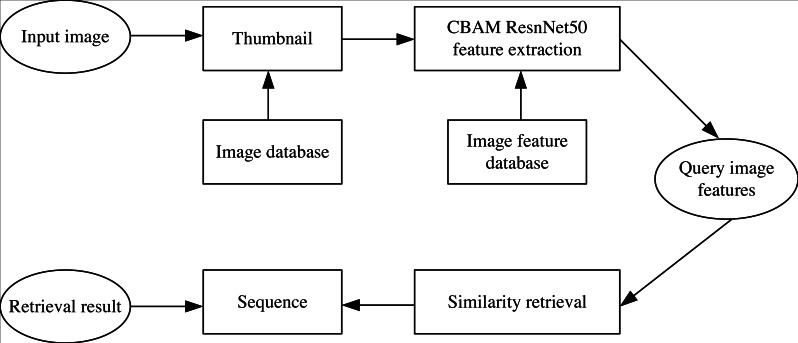
Flow chart of shadow image retrieval model.

The specific flow of the proposed shadow play image retrieval model is shown in Fig. 1. CBAM-ResNet50 network is taken to extract the features of shadow image data sets. Thumbnails are input into the system, the eigenvalues of the search images are removed, compared with all the shadow image features in the data collection, and the obtained image similarity is sorted. The top six photos with similarities are selected. The process can be divided into two parts: online and offline. The offline part extracts features from the shadow image database and generates an image feature database. At the same time, online partial real-time extraction of shadow play image features and offline partial extraction of image features match similarity.

### CBAM-ResNet50

**Figure 2:**
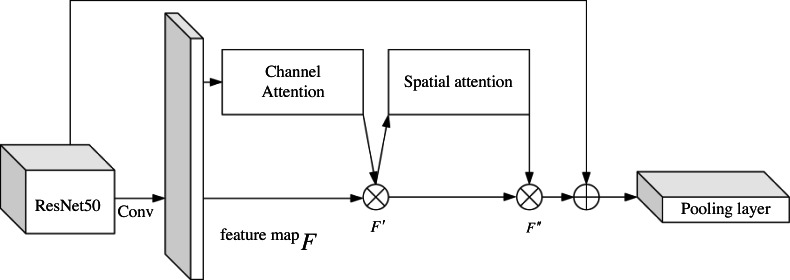
CBAM-ResNet50 structure diagram.

The planned CBAM-ResNet50 network’s organizational structure is shown in Fig. 2. ResNet50’s final convolution layer and pooling layer are connected by the CBAM mechanism, which can extract more local and global features and details from the shadow image while raising network parameters, boosting network robustness, obtaining the necessary feature data from the shadow play image, and enhancing retrieval accuracy.

Among them, *F* represents the feature map, *F*′ represents the feature map after channel attention extraction, and *F*″ represents the feature map after spatial attention extraction.

#### ResNet50

The early algorithm based on feature extraction has a limited ability to express features, and its retrieval effect needs to be improved. The computational cost has also increased significantly with the increasing complexity of neural networks. Using ResNet can reduce the training burden, increase the depth of the neural network, speed up its convergence, and improve the speed and accuracy of searching shadow images. The residual mapping is defined as:

(1) \begin{eqnarray*}F \left( x \right) =H \left( x \right) -x\end{eqnarray*}

where, *x* represents the input value, that is, the feature mapping of the ResNet output of the previous layer; $H \left( x \right) $ represents the observed value of ResNet; $F \left( x \right) $ represents the residual mapping. ResNet comprises stacked residual blocks, and a short circuit mechanism is used to avoid gradient disappearance and explosion. Identity mapping solves the problem of over-fitting caused by increasing network depth, and the network performance will not be reduced. According to the network depth, ResNet can be divided into ResNet 18, 34, 50, 101 and 152. Among them, ResNet 50 has an appropriate network depth, which can achieve faster speed while maintaining high accuracy. Therefore, ResNet 50 is selected as the primary network of the shadow image retrieval model.

#### CBAM

The single ResNet50 network lacks pertinence to the feature structure of shadow puppets, and its ability to distinguish similar shadow puppets is lacking. CBAM is a lightweight general attention module (Nie et al., 2019) for feed-forward CNN. This module obtains attention graphs from different dimensions and performs adaptive feature optimization. Channel attention assigns features according to the input feature map by knowing the importance of each part of the image, focusing on the target in space and adjusting or getting the weight. CBAM is composed of the channel and spatial attention modules arranged in series, and its calculation formula is as follows:

(2) \begin{eqnarray*}Mc(F)=\sigma \left( MLP(Avgpool(F)) \right) +MLP \left( Maxpool(F) \right) \end{eqnarray*}

(3) \begin{eqnarray*}Ms(F)=\sigma ({f}^{7\times 7}(Concat(Avgpool(F),Maxpool(F))))\end{eqnarray*}

(4) \begin{eqnarray*}{F}^{{^{\prime}}}=MC \left( F \right) \otimes F\end{eqnarray*}

(5) \begin{eqnarray*}{F}^{{^{\prime\prime}}}=MS \left( {F}^{{^{\prime}}} \right) \otimes {F}^{{^{\prime}}}\end{eqnarray*}

where *F* is the input image; *MC* is the channel attention weight; *F*′ is the adjusted feature map for channel attention; *MS* is the spatial attention weight; *F*″ is the feature map adjusted for spatial awareness; *σ* is the sigmoid activation function; *f* is the convolution kernel size; *MLP* is the shared full connection layer transformation; *Avgpool* is the pooling of average value; *Maxpool* is the pooling of maximum value; *Concat* represents a splicing operation and ⊗ is the multiplication of the corresponding array elements.

#### Loss function

The loss function determines the direction of gradient decline in the backpropagation process. Continuous training and optimization minimize the loss function value, and finally, the optimal network model (Tolpadi et al., 2022) is obtained. Shadow play image retrieval is a nonlinear regression problem. The mean square error (MSE) is used as the loss function, and the formula is as follows:

(6) \begin{eqnarray*}MSE= \frac{1}{n} \sum _{i=1}^{n}{ \left( di-Di \right) }^{2}\end{eqnarray*}

where *n* represents the characteristics of shadow play images, and respectively represent the characteristics of the original shadow play image *d* and the value of the features *D* of the shadow image processed by CBAM-ResNet50 in *i* the moment.

However, the network converges prematurely and falls into the local optimum, which leads to low retrieval accuracy due to the little difference between the detail features of the shadow image and the inconspicuous gradient change. We propose an improved loss function; the compensation error is calculated by weighting the mean square error and Pearson distance and establishing a loss function under double constraints.

The Pearson correlation coefficient measures the correlation between different features (Panrawee, Shen & Sakdirat, 2022). The correlation degree is calculated by covariance function, then divided by standard deviation to unify the variables to the same order of magnitude to avoid the oscillation of loss function caused by the difference between features, which will affect the learning ability of the network. It can be used to measure the correlation degree between the original and CBAM-ResNet50 processed shadow image features, and its calculation formula is:

(7) \begin{eqnarray*}\rho d,D= \frac{\sum _{i=1}^{n} \left( di-\overline{d} \right) \left( Di-\overline{D} \right) }{\sqrt{\sum _{i=1}^{n}{ \left( di-\overline{d} \right) }^{2}}\sqrt{\sum _{i=1}^{n}{ \left( Di-\overline{D} \right) }^{2}}} \end{eqnarray*}

where $\overline{d}$ and $\overline{D}$ respectively represents the mean value of original shadow play image feature *d* andfeature *D*after CBAM-ResNet50 processing, respectively; *ρd*, *D* represents the Pearson correlation coefficient between *d* and *D*, the range of which is (−1,1). The larger the coefficient, the higher the correlation degree. While the smaller the loss function is, the better the model is, so Pearson distance 1 − *ρd*, *D* is used as a part of the loss function, and the weighted sum with MSE is used as the loss function. The calculation is shown in Formula (8).

(8) \begin{eqnarray*}L=\lambda 1MSE+\lambda 2(1-\rho d,D)\end{eqnarray*}

where *λ*1 and *λ*2 are the weight coefficient.

On the premise that the magnitude of MSE and Pearson distance is consistent, different weights are allocated for weighted summation so that the influence of the two on the gradient is constant, and the gradient change tends to a single loss function so that the network can learn various characteristics. The weight is decided based on the influence of each assessment indicator by the entropy method’s guiding concept. The changes in mean square error and Pearson distance loss in the training process are observed, respectively, and the weights are distributed according to the changes. The smaller the loss changes, the lower the weight, and the larger the loss changes. Since the degree of difference between them equals the weight coefficient. *λ*1 and *λ*2 are both set 1. When the loss function is minimized by gradient descent, the gradient change can be more apparent that the CBAM-ResNet50 network can jump out of the local optimum in the gradient descent process, thus improving the retrieval accuracy.

## IoT System Deployment

**Figure 3:**
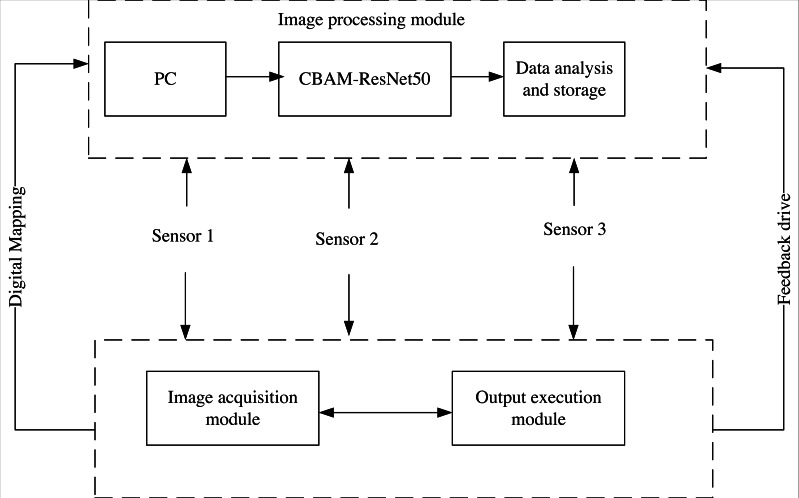
IoT system structure.

IoT system is a technology based on the Internet to connect many low-power interactive devices, which can realize real-time data transmission and dynamic monitoring (Gaurav & Atulya, 2022). This study integrates the CBAM-ResNet50 shadow image retrieval model into the IoT system to achieve more effective shadow image retrieval; Fig. 3 depicts its basic architecture.

(1) Image acquisition module: obtains the shadow image and convert it into a computer-readable data stream, which mainly includes a camera, photo light source, image acquisition card, etc.

(2) Image processing module: includes PC, CBAM-ResNet50 image retrieval model, storage and analysis. Firstly, the PC inputs the retrieval images and instructions, then CBAM-ResNet50 retrieves the received images. Finally, it stores and analyzes the retrieval results and feeds them back to the output execution module.

(3) Sensor: it is mainly responsible for connecting the sending signals of all modules in the system and completing the integration of shadow image features. In this article, the sensor sensitivity is set to 68D, the maximum output frame rate is set to 35 Hz, and the external interface types are the Lan interface, USB interface, 3IO interface and RS232 interface to ensure the sensing range.

(4) Output execution module: the Siemens CPU1214C was selected as the controller of the executive terminal to help people remotely control the model to retrieve images and output the results.

## Experiment and Analysis

The training environment of the model was Windows 10 Home Chinese Edition, Intel(R)Core(TM)i7-5500U processor, 8.00 GB memory, and Ubuntu version of the virtual machine operating system is 18.04.5LTS. PyCharm 2021.1.1 was used for the experiment. Pytorch was a deep learning tool, and the Web framework was Flask.

**Figure 4:**
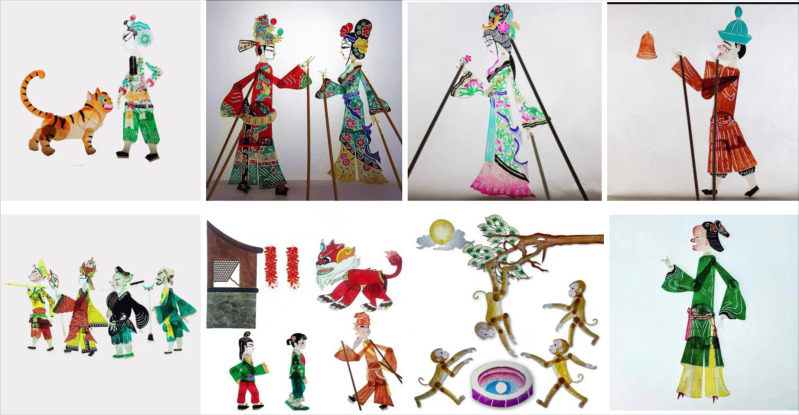
Sample data set.

The shadow image data set constructed in this article is obtained from Baidu, Weibo and other websites, with 500 color images. Each image contains cultural elements of different styles. Manually screen images with rich style information, and enhance the data. After applying various operations like flipping, rotating, cropping, deforming, and scaling, 2,500 shadow play images were created from the collected images. However, this is inconsistent because an image size that is too large or too small will affect the system’s performance or the recognition accuracy. Under the condition of keeping the characteristics of the image unchanged, the height of the shadow play image is fixed at 200 pixels by using the high-priority thumbnail algorithm, and the image’s width is scaled proportionally to the thumbnail according to the original embodiment. Figure 4 shows some pictures in the data set.

### Model training and retrieval performance

**Figure 5:**
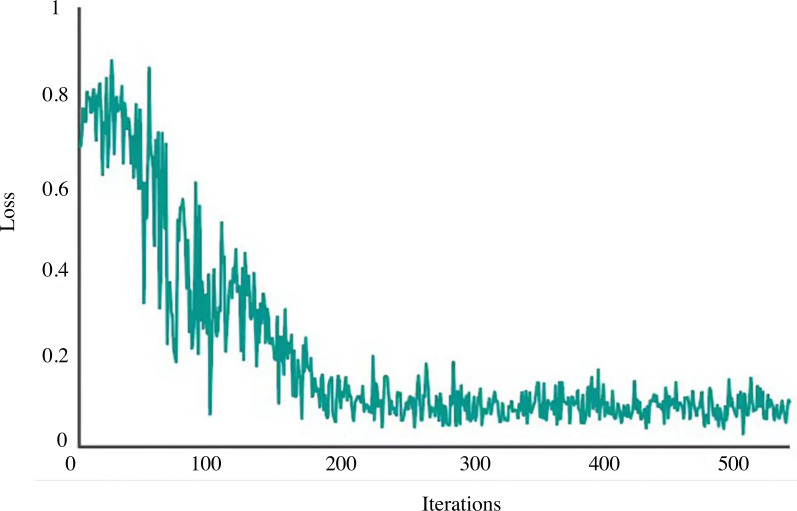
Loss curve.

The network is trained using the shadow play picture data set created in the previous section for 500 iterations to assess this model’s retrieval capability for photos of shadow plays. Figure 5 displays the related Loss curve.

**Figure 6:**
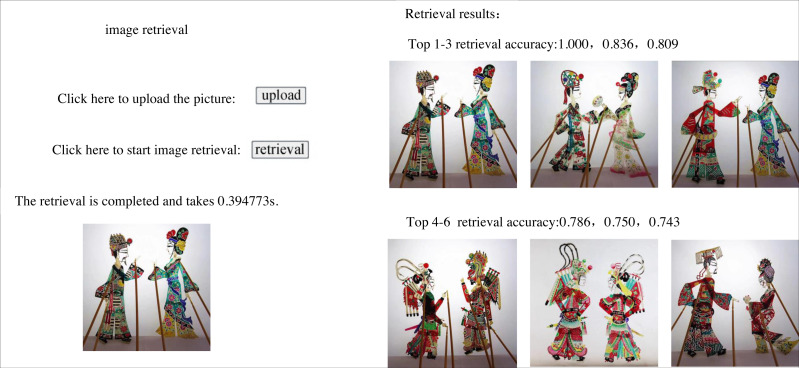
Retrieval effect of this model.

The loss curve of the model used in this study is in a state of rapid decline after 0–100 iterations, and after 200 iterations or so, the loss has stabilised. As a result, this model is robust and has good convergence speed. 300 shadow puppets are selected from the data set and input into the established image retrieval model individually. The depth features of the images are extracted and compared with the shadow puppets features in the image feature database; the similarity is calculated, and the results are output in descending order to obtain the retrieval accuracy of the first six images. Figure 6 shows the retrieval effect of shadow plays images by this model. The first six images found share similar artistic traits, which can be used further to establish the semantic relationship between related shadow play images, contribute to knowledge and information exchange among various shadow play culture research institutes, establish a shadow play culture database, and investigate the internal relationships between shadow play cultures.

 Among the 300 shadow puppets, eight images were not retrieved because the retrieval speed was improved at the cost of partial retrieval accuracy.

### Comparison of different models

To verify the effectiveness of this model on the shadow image data set, different CNN models are used to compare the retrieval accuracy of shadow images. The comparison models are Alex Net (Lu & Wan, 2022), VGG16 (Abdulaziz, 2022), Google Net (Yuan & Kan, 2022) and EfficientNet (Xi & Liu, 2022). The test results are shown in Table 1.

From Table 1, the retrieval accuracy of this model for shadow play images is higher than that of other models. It is most suitable for feature extraction as a backbone network. Compared with other models, the AlexNet network has shallow layers and poor performance; GooLeNet model with Inception structure can perform convolution and pooling operations on the input image in parallel to obtain other information on the input image and combine all the results to get more accurate image features; EfficientNet has achieved high retrieval accuracy, but the network layers are too deep, which quickly leads to over-fitting. However, the network layer of our model is moderate. Meanwhile, the attention mechanism is added, so the ability to extract shadow image features is more vital, and the highest retrieval accuracy is achieved.

**Table 1:** Comparison of retrieval accuracy of different models.

Model	Accuracy of the first image retrieval	Average retrieval accuracy of the first six images
AlexNet	73.1	76.8
VGG16	83.1	86.3
GoogLeNet	74.7	88.6
EfficientNet	80.5	90.1
Model of this article	1.00	92.5

### Ablation experiment

An ablation experiment was created to evaluate the optimization effect of CBAM on the model and investigate the influence of the attention mechanism on the model’s capacity to retrieve information. The specific settings are (1) ResNet50, only ResNet50 is used as feature extractor; (2) ResNet50+CA, based on ResNet50, a channel attention module is added; (3) ResNet50+SA, based on ResNet50, the spatial attention module is added; (4) ResNet50+ CBAM, based on ResNet50, a serial channel and spatial attention module are added; (5) CBAM+ResNet50+CBAM, adding serial channels and spatial attention modules before and after ResNet50. The results are shown in Table 2.

**Table 2:** Comparison of retrieval accuracy of different ResNet50 models.

Model	Accuracy of the first image retrieval	Average retrieval accuracy of the first six images
ResNet50	83.4	86.8
ResNet50+CA	83.8	89.1
ResNet50+SA	84.3	90.2
Model of this article	1.00	92.5
CBAM+ResNet50+CBAM	75.6	82.9

From Table 2, the retrieval accuracy of this model is higher than that of other models, mainly because a single serial channel and spatial attention can help the network extract more detailed features. Meanwhile, improving the loss function has further enhanced the retrieval ability of the model. Therefore, the proposed model has achieved better progress on ResNet50 and can be used for effective shadow play image retrieval.

## Conclusion

This work offers a shadow play image retrieval model based on CBAM-ResNet50 to enable efficient and accurate retrieval and analysis of shadow play images and widen traditional Chinese culture’s transmission channels. The CBAM mechanism is introduced into the ResNet50 network, which makes the image retrieval model assign attention weight to the retrieval features when recognizing images, thus improving the recognition rate of the model and shortening the retrieval time. The improved loss function can help the model extract the shadow image features more effectively, giving the feature descriptor a strong recognition ability. The model was deployed to the IoT system for more efficient shadow image retrieval. Experiments show that the average retrieval accuracy of the first six shadow puppets is 92.5%, which can realize accurate shadow play image retrieval and provide a new direction for the spread of Shaanxi traditional culture. Although the model in this article can achieve accurate and efficient shadow play image retrieval, there are too few image styles in the data set, and it is difficult to guarantee the effect of the model when applied to practice. Therefore, in the next step, we will further expand the dataset, improve the stability of the model, and ensure the accuracy and stability of the model in practical application.

##  Supplemental Information

10.7717/peerj-cs.1330/supp-1 Supplemental Information 1 Code.

## Additional Information and Declarations

### Data Deposition

The following information was supplied regarding data availability: The code is available in the Supplementary File. The data is available at Zenodo: tzw. (2023). Chinese Cultural data [Data set]. Zenodo. https://doi.org/10.5281/zenodo.7622122.
